# Visual and olfactory signals of conspecifics induce emotional contagion in mice

**DOI:** 10.1098/rspb.2024.1815

**Published:** 2024-12-11

**Authors:** Madoka Nakamura, Kensaku Nomoto, Kazutaka Mogi, Tsuyoshi Koide, Takefumi Kikusui

**Affiliations:** ^1^ Department of Animal Science and Biotechnology, School of Veterinary Medicine, Azabu University, 1-17-71, Fuchinobe, Sagamihara, Kanagawa 252-5201, Japan; ^2^ Department of Ultrastructural Research, National Institute of Neuroscience, National Center of Neurology and Psychiatry, 4-1-1 Ogawahigashi-machi, Kodaira, Tokyo 187-8553, Japan; ^3^ Department of Physiology, Dokkyo Medical University School of Medicine, 880 Kitakobayashi, Mibu, Shimotsugagun, Tochigi 321-0293, Japan; ^4^ Mouse Genomics Resource Laboratory, National Institute of Genetics, 1111 Yata, Mishima, Shizuoka 411-8540, Japan; ^5^ Graduate Institute for Advanced Studies, SOKENDAI, 1111 Yata, Mishima, Shizuoka 411-8540, Japan

**Keywords:** emotional contagion, social signal, empathy

## Abstract

Emotional contagion occurs in many animals, including rodents. To determine the social signals of emotional state-matching between individuals in mice, we conducted an empirical laboratory experiment using visual, olfactory and auditory stimuli. The Japanese wild-derived mouse strain MSM/Ms (MSM) was tested as observers, since our initial experiments indicated that MSM mice showed higher sensitivity to others’ pain compared with the laboratory strain C57BL/6J (B6). MSM observers were shown footage of an unfamiliar B6 mouse receiving painful foot shocks via a screen. For olfactory stimuli, one of the following was presented during observation: (i) urine collected from a shocked B6 mouse, (ii) urine collected from an unshocked B6 mouse, or (iii) reverse osmosis water. Consequently, MSM mice observing the footage with urine from shocked mice demonstrated significantly higher fear-induced freezing behaviour than in the other two conditions. Regarding visual and auditory stimuli, observing the pixelated video clip was significantly associated with reduced freeze responses, whereas blocking auditory cues did not affect the duration of freezing. These results provide clear-cut evidence that multiple cues, including olfactory and visual information, are sufficient social signals for emotional contagion in mice.

## 1. Introduction

Emotional contagion, the simplest form of empathy defined as a quick emotional state-matching between individuals [1,2], is essential for the regulation of social interactions and cooperations. It is generally believed that the selection pressure to evolve rapid emotional connectedness began in the context of parental nurturance for altricial offspring [3,4]. Since young organisms require food and protection, parents need to pay close attention to the emotional signals from their vulnerable infants. Consequently, the underlying neural mechanisms for responding to offspring seem to be conserved across species [5]. However, several studies have shown that animals, including rodents, exhibit robust emotional contagion in response to the distress of others, not only among kin but also among unrelated individuals such as cohabiting animals [4,6–8].

Emotional responses to displays of emotion in others are well established in laboratory experiments using various animals ranging from primates [9,10] to rodents [7,11] and avian species [12,13], suggesting that a basis for empathy may be widely conserved beyond species. Rodents, including mice and rats, exhibit social modulation of pain [7], consolation [14] and prosocial behaviour [11,15]. A fundamental feature of empathy is therefore conserved from rodents to humans, as human performance in similar observational fear processes correlates with trait measures of empathy [16–19]. These rodent and human studies suggest that emotional empathy in mammals can be studied through the behaviour of rodents in response to observing conspecific distress.

A behavioural mouse model is commonly used to investigate the neural mechanisms underlying empathy. In observational fear conditioning, a rodent behavioural model for assessing empathic fear [20–23], a mouse shows fear-induced freezing behaviour after observing a conspecific receive electric foot shocks. When visual inputs are blocked by replacing the transparent partition with an opaque black panel, the fear response of the observer is significantly reduced [21]. This result indicates that visual cues are involved in the induction of freezing behaviour.

Classical laboratory mouse strains have an extensive history of experimental use and are excellent tools for genetics and other scientific studies [24]. However, several behavioural responses have been altered or sometimes attenuated in laboratory strains owing to active selection for domestication [25–28]. Thus, wild-derived mice retaining several behavioural characteristics of wild mice have been introduced into research laboratories, many of them established as inbred strains [29]. A Japanese wild-derived inbred mouse strain, MSM/Ms (MSM), established from Japanese wild mice collected in 1978 in Mishima, Japan, exhibits several strain-specific characteristics, such as higher spontaneous activity in the home cage, increased freezing and grooming behaviours in novel situations and difficulty in habituation to novelty [28,30,31].

In this study, we first examined whether emotional contagion was more strongly preserved in MSM mice compared with the standard laboratory strain C57BL/6J (B6). The freezing behaviour of B6 and MSM observers witnessing unfamiliar demonstrators receiving repetitive foot shocks was statistically compared. Second, we conducted empirical laboratory experiments to determine the social signals of emotional state-matching between individuals in mice. While the findings support that the emotional process occurs in rodents [20,22,23], it is still unknown what specific triggers form the basal layer of empathy. Since olfactory, visual and auditory signals are considered the three main sensory modalities in animals, we focused on these three as possible signals for emotional contagion and identified which were the essential signals for the simplest form of empathy.

## 2. Material and methods

### (a) Animals

In this study, 8–17-week-old adult male MSM and B6 mice were used. After weaning, mice were socially housed in same-sex, same-strain groups of 2–6 in standard polycarbonate mouse cages (220 × 320 × 135 mm or 140 × 320 × 140 mm, Natsume Seisakusho Co., Tokyo, Japan) at a room temperature of 23 ± 2°C. They were kept on a 12 h : 12 h light–dark cycle with food and water *ad libitum*. All animal experiments were conducted under protocols approved by the Institutional Committee for Animal Care and Use of the National Institute of Genetics (NIG), Shizuoka, Japan (permit numbers 26-9, 27-11 and 28-5).

### (b) Experimental paradigm

#### (i) Observational fear test

**Figure 1.**
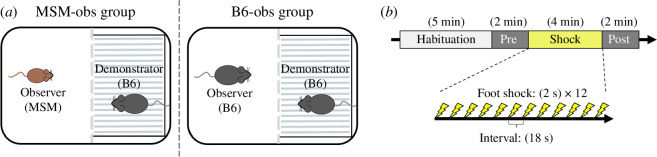
Experimental set-up and time schedule. (*a*) Top views of the experimental set-up. The experiments were conducted in two groups: (left) MSM observers with B6 demonstrators and (right) B6 observers with B6 demonstrators (*n* = 10 for each group). (*b*) All observational fear experiments were conducted with this time schedule: a 5 min habituation period, a 2 min pre-period, a 4 min shock period and a 2 min post-period.

Demonstrators (B6) and observers (MSM or B6) were individually placed in apparatus chambers partitioned by a transparent plastic perforated plate (O'Hara ＆Co., Tokyo, Japan; figure 1*a*). To ensure consistency in the experimental paradigm, we restricted the demonstrator strain to B6. After a 5 min habituation period and a 2 min pre-period, a 2 s 0.20 mA foot shock was delivered every 20 s for 4 min (figure 1*b*) to the demonstrator through a stainless-steel grid floor. Observers were able to witness an unfamiliar, non-cagemate demonstrator receiving repetitive foot shocks.

#### (ii) Social-signal-exposure experiment

**Figure 2.**
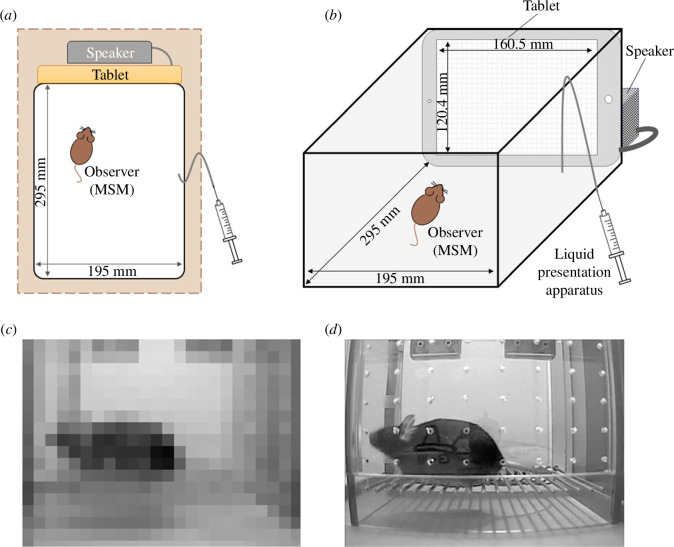
Experimental apparatus and footage. (*a*) Top view of the experimental set-up. (*b*) Diagram of the apparatus used for the social-signal-exposure experiment. The range of the fundamental frequency of the speaker was from 68 to 13 kHz. Observers were shown the footage on the tablet screen in either (*c*) pixelated or (*d*) unprocessed form.

MSM observers were shown footage on an iPad mini 3 (200 × 133.7 mm, Apple, USA) tablet of an unfamiliar B6 mouse receiving 0.99 mA painful foot shocks. The apparatus was covered with cardboard to make the surroundings invisible (figure 2*a*). Presenting footage through tablets enables the presentation of precisely the same stimuli to each individual on every occasion. The video clip for observers was set to 13 min, according to the time schedule of the observational fear test (figure 1*b*), and used for all MSM observers. Using a tablet is effective in blocking olfactory cues and unifying visual and auditory cues. We then artificially presented each signal individually. The sound of the footage was presented by a SoundLink Mini speaker (BOSE Co., USA) connected to the iPad (figure 2*a*,*b*). Previous research on ultrasonic vocalizations (USVs) has shown that mice do not emit USVs in response to aversive stimuli, whereas adult rats emit a 22 kHz vocalization in anticipation of inescapable aversive stimuli [32]. Based on the results of this study, we recorded and presented the video clip only in the audio frequency range.

To identify which sensory modalities are the essential signals for the simplest form of empathy, the percentage of time spent freezing by observers was compared across conditions (see table 1). First, we examined whether olfactory cues were necessary for emotional contagion. As soon as the 4 min shock period of the footage started, one of the following stimuli was presented by the experimenter through a polyethylene tube: (i) urine collected from a shocked B6 mouse, (ii) urine collected from an unshocked B6 mouse, or (iii) reverse osmosis (RO) water. Mouse urine (i) and (ii) were collected in a room separate from the experimental room just before the tests. The tube on the side wall of the apparatus (Natsume Seisakusho Co., Tokyo, Japan) had an inner diameter of 0.8 mm, with a 21G needle and a 1 ml disposable syringe (Terumo Corporation, Tokyo, Japan), and 0.1–0.2 ml of the liquid was presented at each session.

**Table 1.** Experimental conditions and presented signals in the social-signal-exposure experiment. Iitalic type indicates the signals that compare multiple conditions. RO, reverse osmosis.

	olfactory signals	visual signals	auditory signals
experiment ii-a	*urine: shocked* *urine: unshocked* *RO water*	unprocessed	with sound
experiment ii-b	urine: shocked	*pixelated* *unprocessed*	with sound
experiment ii-c	urine: shocked	unprocessed	*with sound* *without sound*

Second, we investigated whether visual signals were essential for empathic responses. The footage of a B6 mouse receiving foot shocks was pixelated in 20-pixel units (figure 2*c*) using the video editing software Filmora (Wondershare Technology Co., Shenzhen, China). Either the pixelated or an unprocessed video clip (figure 2*d*; electronic supplementary material, video S1) with sound was presented to the observers while using urine collected from a shocked B6 mouse as olfactory cue.

Finally, we examined whether auditory cues were required for emotional state-matching. Observers witnessed the unprocessed video clip with or without sound. We compared the freezing behaviour of observers in the two groups to determine whether the presence or absence of sound affected their freezing behaviour, while using urine collected from a shocked B6 mouse as olfactory cue.

### (c) Behavioural scoring and analysis

The entire test sessions were recorded by a video camera (JVC GZ-R300-T, Victor Company of Japan) mounted on the apparatus. The total duration of freezing behaviour, defined as the complete absence of body movements excluding respiration [33], was scored in seconds during the 13 min sessions. Freezing scores were calculated as the percentage of time during each period that the mice spent freezing. JMP (version 17.2.0, SAS Institute, Cary, NC, USA) was used to perform all statistical tests. All values were expressed as the mean ± s.e., and *p* < 0.05 was considered statistically significant. Comparisons involving more than two groups or conditions were calculated using two-way repeated measures ANOVA followed by Tukey’s HSD *post hoc* tests. A comprehensive table detailing the *F*-values, *p*-values (including main effects, interactions and *post hoc* test results) is presented in the supplementary material (electronic supplementary material, table S1).

## 3. Results

### (a) MSM mice showed higher sensitivity to others’ pain compared with B6 mice

**Figure 3.**
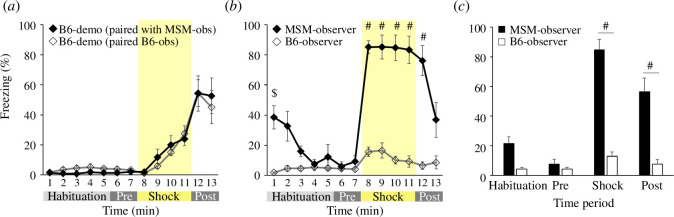
MSM mice demonstrated significantly higher fear-induced freezing behaviours when observing others’ pain compared with B6 observers. (*a*) Quantification of freezing behaviour with a 1 min resolution in B6 demonstrators paired with MSM observers (*n* = 10) and B6 demonstrators paired with B6 observers (*n* = 10). There was no difference in the freezing time between the two groups. (*b*) Quantification of freezing behaviour with a 1 min resolution in MSM observers (*n* = 10) and B6 observers (*n* = 10). MSM observers exhibited significantly higher freezing behaviour during the ‘shock’ and ‘post’ periods. (*c*) Quantification of freezing behaviour in MSM observers (*n* = 10) and B6 observers (*n* = 10) divided into four time periods. ^#^*p* < 0.0001, ^$^*p* = 0.0023.

In the observational fear test, we compared the freezing behaviour of MSM and B6 observers to investigate differences in sensitivity to others’ pain between the two strains. Freezing rates exhibited a similar pattern across time bins for both groups of demonstrators (two-way ANOVA, group × time bin, *F*_12,216_ = 0.3515, *p* = 0.978, figure 3*a*). The two-way ANOVA of the observer’s freezing responses during the test session showed group × time bin interaction (*F*_12,216_ = 17.8991, *p* < 0.0001; figure 3*b*). The *post hoc* analyses indicated that MSM observers froze significantly more than B6 observers at the first minute (*p* = 0.0023) and from 8–12 min (all *p* < 0.0001), whereas no significant difference was detected between the groups in the 2 min before the shock delivery (figure 3*b*). Statistical analysis of the four time periods revealed that MSM observers significantly froze in the 4 min shock period and the 2 min post-period, as shown by two-way ANOVA (group × time period, *F*_3,54_ = 22.3692, *p* < 0.0001; figure 3*c*) and *post ho*c test (both *p* < 0.0001; figure 3*c*). These results indicate that the wild-derived mouse strain MSM demonstrates significantly higher fear-induced freezing behaviour when observing others’ pain compared with B6.

### (b) Olfactory and visual information are required for emotional contagion

To determine which sensory modalities are essential signals for the simplest form of empathy, we compared freezing behaviour among observers across various conditions (table 1). Given that MSM mice had been shown to be more sensitive to the emotions of others than B6 mice in the observational fear test (figure 3*b*,*c*), we used MSM mice as observers to identify the signals necessary for emotional contagion. In rodents, olfactory information is considered the most crucial sensory modality. Therefore, in this experiment, olfaction was initially tested, and it was subsequently determined that mice exposed to urine collected during shock showed significantly increased freezing behaviour. Using this urine from shocked mice, we then demonstrated the contributions of visual and auditory modalities, respectively.

**Figure 4.**
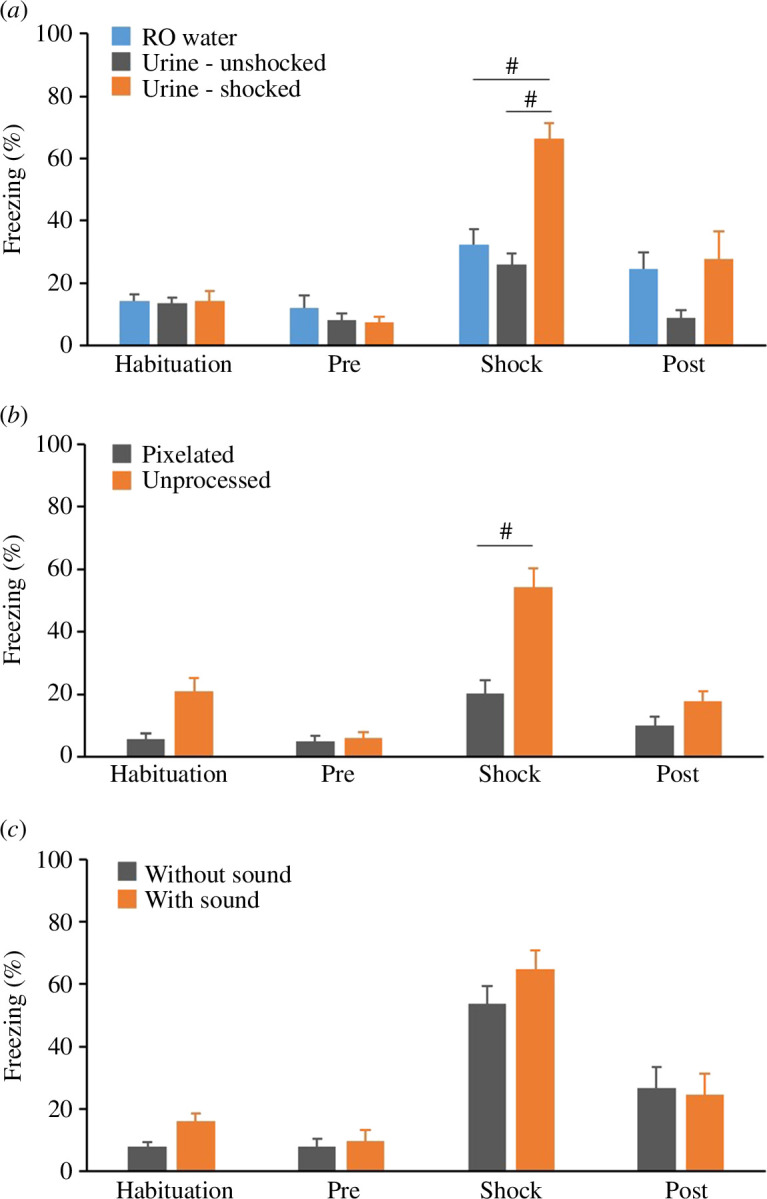
Olfactory and visual cues are required for emotional contagion in mice. (*a*) Quantification of freezing behaviour in MSM observers in the three different conditions of olfactory signals: Reverse osmosis (RO) water (*n* = 10), urine: unshocked (*n* = 10), urine: shocked (*n* = 8). MSM mice observing the footage while exposed to shocked urine demonstrated significantly higher freezing behaviour in the shock period than MSM mice in the other two liquid conditions. (*b*) Quantification of freezing behaviour in MSM observers in the two different conditions of visual signals: pixelated (*n* = 9) and unprocessed (*n* = 7). MSM mice observing the raw video clip of a B6 mouse receiving foot shocks clearly showed significantly higher freezing behaviour during the shock period. (*c*) Quantification of freezing behaviour in MSM observers in the two different conditions of auditory signals: without sound (*n* = 9) and with sound (*n* = 7). There was no difference in the freezing time between the two groups. Two-way ANOVA with Tukey’s *post hoc* test. ^#^*p* < 0.0001.

The two-way ANOVA of the observer’s freezing responses during the olfactory signals test session showed condition × time period interaction (*F*_6,75_ = 4.3051, *p* = 0.0009; figure 4*a*). The *post hoc* analyses indicated that MSM mice observing the footage hile exposed to urine from shocked mice demonstrated significantly higher freezing behaviour than those in the other two liquid conditions (both *p* < 0.0001; figure 4*a*). There was no significant difference between the pre-period and shock period both in the RO water condition (*p* = 0.1682; represented by the blue bars in figure 4*a*) and unshocked urine condition (*p* = 0.3425; represented by the black bars in figure 4*a*), suggesting that the urine collected from a shocked mouse may contain substances that induce emotional state-matching in mice.

Compared with the pixelated footage condition, MSM mice observing the unprocessed video clip of B6 mouse receiving foot shocks clearly showed significantly higher freezing behaviour during the shock period, as shown by two-way ANOVA (condition × time period, *F*_3,42_ = 7.4386, *p* = 0.0004; figure 4*b*) and *post hoc* test (*p* < 0.0001; figure 4*b*). There was no statistically significant difference between the pre-period and shock period in the pixelated footage condition (*p* = 0.0573; represented by the black bars in figure 4*b*), suggesting that mice may need to visually recognize another individual’s form or movement for the emotional contagion to occur, whereas blocking auditory cues did not affect the duration of freezing (two-way ANOVA, *F*_3,42.54_ = 0.1643, *p* = 0.9198; figure 4*c*).

## 4. Discussion

We found that the wild-derived inbred strain MSM has higher sensitivity to others’ pain compared with the standard experimental strain B6. This suggests that the function of emotional contagion is more strongly preserved in the wild-derived mouse strain than in the laboratory strain. According to the social-signal-exposure experiment in this study, MSM mice showed the highest emotional reactivity to others when urine collected from a mouse receiving electric foot shocks was presented together with visual and auditory cues during the observation.

While our study did not investigate the specific components in the urine, there is a possibility that the urine of mice showing a fear response contains certain substances not normally present in natural urine. It has been known for some time that the male murine urinary pheromone 2-*sec*-butyl-4,5-dihydrothiazole (SBT) is a substance that induces inter-male aggression. However, an earlier study suggested that SBT also conveys predator-specific alarm information to other conspecifics [34]. In this study, SBT may have been released into the B6 mice urine as an automatic reaction to receiving foot shocks, significantly increasing the observers’ freezing behaviour owing to the alarm pheromone artificially presented through the apparatus.

Many previous studies on emotional contagion have aimed to identify the essential elements by systematically blocking each potential contributing factor. For example, in the acetic acid-induced writhing test, a mouse behavioural model for assessing nociceptive sensitivity, the observing mouse’s sensitivity to noxious stimuli increases when exposed to a cage mate displaying pain behaviour [7]. Although this pain contagion persists even when sensory input is individually blocked by disrupting the mouse’s olfactory epithelium, the possibility of pheromonal communication cannot be ruled out, as axonal transport from the vomeronasal organ to the accessory olfactory bulb remains intact. Therefore, this study clarified the extent to which each element contributes to emotional contagion. The presentation of each signal through a tablet, liquid tube, and speaker in this study allowed precise and consistent presentation of olfactory, visual and auditory stimuli, enhancing the accuracy and reproducibility of the experimental conditions.

Our data clearly revealed that emotional contagion in the wild-derived strain occurs through the perception of emotional states of others via two-dimensional footage. Additionally, observers' freezing responses weakened unless they were able to visually recognize the movement or morphology of individuals. This suggests that visual information is a significant signal for mice to detect another’s emotion, aligning with the findings from previous studies [7,35]. Although the increased freezing behaviour of observers when presented a pixelated video (figure 2*c*) did not reach statistical significance compared with the pre-period, it suggests that the observers were at least partially responsive to the pixelated visual stimuli. While this study demonstrated the significance of visual information, it was unable to discern whether the specific movement patterns of conspecifics or the visual characteristics of the moving stimuli were primarily responsible for inducing freezing behaviour. Future research could elucidate this issue by using experimental paradigms such as the presentation of multiple static images of freezing conspecifics to assess the role of shape recognition, or by presenting highly abstracted moving stimuli (e.g. black spheres) that mimic mouse movements to examine the importance of motion perception. In the auditory signal comparison test, we did not detect an effect under the current experimental conditions, suggesting that auditory cues were not necessary information in this context. However, this does not necessarily mean that auditory information is meaningless. Since mice produce vocalizations to communicate with others, individuals may select the appropriate information sources according to the environment. One limitation of this study is that the experimental paradigm used may have confounded fear and pain responses in the transmitted signals. Future studies should therefore utilize demonstrators that exhibit solely fear responses to isolate the effects of fear transmission.

As previously stated in the introduction, laboratory mouse strains were established with features or behavioural characteristics selected by experimenters to allow easy handling. As a result of extensive inbreeding and genetic selection, B6 mice, a commonly used inbred strain, display a number of behavioural differences such as reduced anxiety compared with wild-derived mouse strains [30]. Conversely, MSM mice, originating from a non-domesticated population, are likely to exhibit greater sensitivity to conspecific fear cues and thus may display more persistent freezing behaviour. B6 demonstrators, owing to the repeated administration of painful stimuli, exhibited avoidance behaviours in response to the stimuli, suggesting that the relative incidence of freezing behaviour may have decreased. Despite the experiments being conducted between different strains in the observational fear test (figure 1*a*), MSM observers exhibited stronger freezing behaviour compared with B6 demonstrators. While a direct comparison between mice of the same strain is needed to draw a definitive conclusion, these results suggest that MSM mice may be particularly sensitive to emotional contagion signals shared among different mouse strains. Furthermore, under the breeding condition, there may no longer be a need to maintain vision and olfaction sufficient to respond to potential threats. It can therefore be concluded that the reduction of innate behaviour in laboratory strains was influenced by strong selection and breeding circumstances. While the experimental advantages of inbred strains including B6 are undeniable, the use of wild-derived strains such as MSM can offer valuable insights into the natural behavioural phenotype.

In conclusion, we have demonstrated that multimodal social cues, including olfactory and visual information, are required to detect another individual’s emotional state. It is generally believed that rodents mainly use olfactory cues in their environment to navigate and interact socially with one another. As we succeeded in independently presenting each signal to demonstrators, it became clear that both olfactory and visual cues are necessary for emotional contagion of pain in mice.

## Data Availability

Data have been deposited in Dryad [36]. Supplementary material is available online [37].
